# Nano-LC with New Hydrophobic Monolith Based on 9-Antracenylmethyl Methacrylate for Biomolecule Separation

**DOI:** 10.3390/ijms252413646

**Published:** 2024-12-20

**Authors:** Cemil Aydoğan, Sarah Alharthi

**Affiliations:** 1 Food Analysis and Research Laboratory, Bingöl University, 12000 Bingöl, Türkiye; 2 Department of Food Engineering, Bingöl University, 12000 Bingöl, Türkiye; 3 Department of Chemistry, Bingöl University, 12000 Bingöl, Türkiye; 4 Department of Chemistry, College of Science, Taif University, Taif P.O. Box 11099, Saudi Arabia; sarah.alharthi@tu.edu.sa

**Keywords:** nano-liquid chromatography, peptide, proteomics, miniaturization, monolith, reversed phase

## Abstract

In this study, new monolithic poly(9-anthracenylmethyl methacrylate-co-trimethylolpropane trimethacrylate (TRIM) columns, referred as ANM monoliths were prepared, for the first time, and were used for the separation media for biomolecules and proteomics analysis by nano-liquid chromatography (nano-LC). Monolithic columns were prepared by in situ polymerization of 9-anthracenylmethyl methacrylate (ANM) and trimethylolpropane trimethacrylate (TRIM) in a fused silica capillary column of 100 µm ID. Polymerization solution was optimized in relation to monomer and porogenic solvent. Scanning electron microscopy (SEM) and chromatographic analyses were performed for the characterization studies of ANM monoliths. The ANM monolith produced more than 46.220 plates/m, and the chromatographic evaluation of the optimized ANM monolith was carried out using homologous alkylbenzenes (ABs) and polyaromatic hydrocarbons (PAHs), allowing both strong hydrophobic and π-π interactions. Run-to-run and column-to-column reproducibility values were found as <2.91% and 2.9–3.2%, respectively. The final monolith was used for biomolecule separation, including both three dipeptides, including Alanine-Tyrosine (Ala-Tyr), Glycine-Phenylalanine (Gly-Phe), and L-carnosine and five standard proteins, including ribonuclease A (RNase A), α-chymotrypsinogen (α-chym), lysozyme (Lys), cytochrome C (Cyt C), and myoglobin (Mb) in order to evaluate its potential. Both peptides and proteins were baseline separated using the developed ANM monolith in nano-LC. The ANM monolith was then applied to the protein and peptide profiling of MCF-7 cell line, which allowed a high-resolution analysis of peptides, providing a high peak capacity.

## 1. Introduction

Advances towards the improvement of low-flow-rate LC in both instrument hardware and methodologies for significant analyses are very crucial in bioanalytical analyses, especially in proteomics [1]. The use of narrow bore columns with the low flow rates of ≤800 nL/min is crucial in modern liquid chromatography. Among other low-flow-rate LC systems, nano-LC is the most common tool for biomolecule separations, representing a universal solution for different analytical separations. The enhanced sensitivity is the main advantage for nano-LC since this is particularly important in the determination of low abundant compounds in limited samples [2]. The narrow bore columns with ≤100 µm IDs are used in nano-LC [3], while nano-LC also holds several options to improve speed and resolution. Although particle-packed columns are commonly used for nano-LC separations, especially in proteomics analyses [4], their preparation and usage in a narrow format (e.g., ≤100 µm ID) is difficult, and those columns are very expensive. Monolithic columns are increasingly utilized in low-flow-rate LC separations, including nano-LC, for a wide range of compounds of small to very large moleculer sizes [2,5,6]. The virtue of monolithic columns includes the ease of in situ column preparation and provides good alternatives with several advantages over particle-packed columns, such as the low carry-over characteristics. The development and use of new narrow columns are very important issues for advanced bioanalytical analyses, including omics analysis such as proteomics [7]. Nano-LC with the use of narrow bore columns is an ideal for use in advanced protein and peptide profiling. For instance, 10.000 different proteins were identified in tumor tissue samples collected from 88 different patients, which allows one to identify relevant proteomic fingerprints [8]. Deep protein and peptide profiling are highly challenging due to the fact that the proteome samples are extremely limited in size [9,10]. There are several challenges in advanced proteomics analysis such as proteome coverage, automation, and robustness. A hundred thousand protein species are in the possibilities of proteoforms. In this sense, a highly sensitive detection of low abundant proteins in the samples needs the use of advanced nano-LC methodologies with new narrow bore columns. A sensitive proteome analysis of extremely limited samples is a highly essential issue, and this needs to use advanced tools to readily analyze the proteoforms and modifications [11]. In this sense, a highly sensitive detection of proteoforms needs the use of new and advanced narrow ID columns. In our previous studies, various narrow bore monolithic columns were prepared and used in nano-LC for protein and peptide profiling [12]. 

The use of a new monomer chemistry for the preparation of new monolithic structures is always refreshing. In this study, we used ANM together with a TRIM and porogenic mixture in order to prepare a new monolith for proteomic analysis. In this sense, a new 9-antracenylmethyl methacrylate-based monolith was prepared and tested for hydrophobic compound and biomolecule separation and potential application in LC–MS proteomic analysis. Since the monolith included the merits of ANM, various parameters, including different amounts of ANM, were investigated. The potential of ANM monolith was demonstrated using a gradient separation of both five standard proteins and three dipeptides, while the further protein and peptide profiling of MCF-7 cell line was performed by applying the developed ANM monolith in nano-LC/UV system.

## 2. Results and Discussion

### 2.1. The Preparation and Characterization of ANM Monolith

The optimization of key variables such as monomer and porogen content as well as optimal temperature for the preparation of new monolith is necessary to produce a robust monolith that could be sufficiently permeable to provide efficient chromatographic separation. In our previous studies, different hydrophobic monolithic columns were prepared and used for protein and peptide profiling [12]. Recently, 2-vinylnapthalene as the main monomer was used for the preparation of the open-tubular columns for intact protein and peptide separation [13], which was not a suitable monomer for monolith preparation due to its several chemical properties such as vinyl group without carboxyl, allowing a thin layer coating of the capillary surface. In the present study, ANM was selected as the main monomer due to its good hydrophobic structure, which may allow a high level of hydrophobic interaction for chromatographic separations. EDMA is mostly used as a crosslinker in monolith preparation. In this study, TRIM, trimethylolpropane trimethacrylate, a trifunctional monomer was considered as the crosslinker due to its reported ability to give rise to macroporous polymers under relevant conditions [14]. This is particularly important for hydrophobic monoliths since macromolecules such as proteins need good flow-through pores. TRIM may also allow good loading capacity for protein and peptide profiling. Monolith porosity also depends on the selection of porogenic solvents, which is a key factor that may lead to the tuning of the monolith porosity. In the present study, several porogenic solvents were tested to optimize the monolith structure such as cyclohexanol /dodecanol, THF/dodecanol, and toluene/dodecanol. Among them, the mixture of THF/dodecanol gave the best results for the monolith preparation according to both hydrodynamic properties and robustness. It was also shown that the ratio between these solvents is very crucial regarding the performance of the ANM monolithic column. The content of polymeric monomers with hydrophobic ligand is often changed in the preparation of hydrophobic monoliths. The content of ANM was changed from 2.7 mg to 16.52 mg (*w*/*w*) in the polymerization mixture while keeping the content of TRIM the same. The further increase in ANM monomer content in the polymerization solution led to the composition of more viscous solution, which was not suitable for the monolith preparation. It was shown that the column permeability was affected, allowing a too-small flow rate. The surface area of the prepared ANM-8 columns was calculated as 288.4 m^2^/g (see Table 1). It was shown that the specific surface area enhanced with the increasing content of ANM in the polymerization solution. The content of TRIM was also increased, allowing the optimized molar ratio with 1.0:3.6 (*v*/*v*) between the ANM and TRIM, respectively.

**Figure 1:**
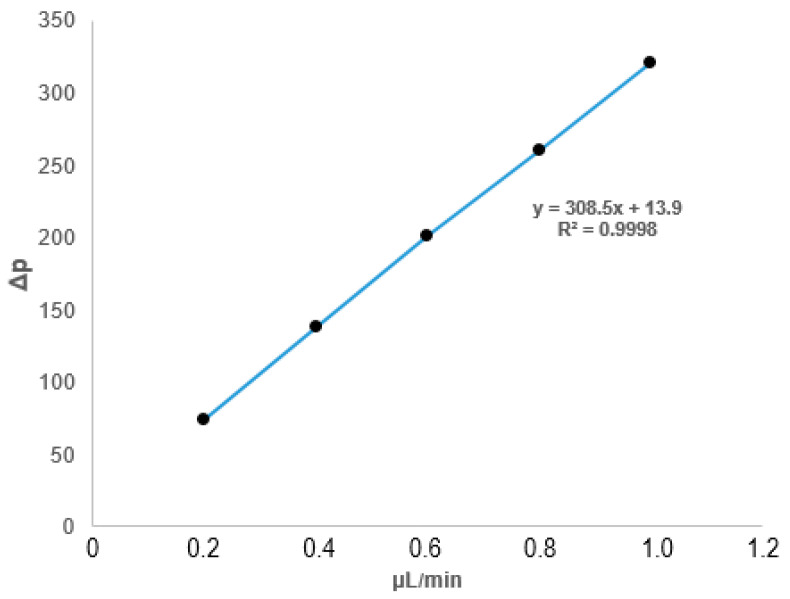
Plot of ANM-8 monolithic column back pressure versus flow rate using ACN:H_2_O (80:20%, *v*/*v*).

This optimized monolith was robust and had good permeability. The linear relationship value was found as R^2^ = 0.9998 (see Figure 1).

**Figure 2:**
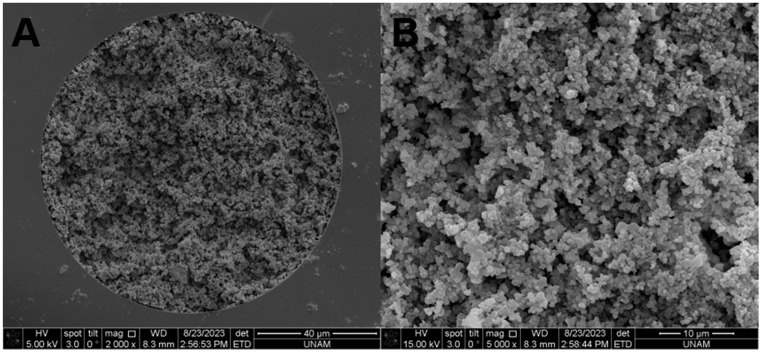
SEM images of ANM-8 monolithic column with the magnification of (**A**) 2000× and (**B**) 5000×.

SEM images of the optimized monolithic column are given in Figure 2 for the ANM-8 monolithic column. While Figure 2A shows the stationary phase incorporated strictly coated on the column inner surface, Figure 2B revealed that both nanoglobules with less than 300 nm could be obtained, and the pore size was about 4 μm. These results prove the good surface area of the developed monolithic column.

Column reproducibility was investigated in terms of the percent relative standard deviations (RSDs) of the *k* values of three test solutes including uracil (*k*_av_ = 0.54), toluene (*k*_av_ = 2.88), and butylbenzene (*k*_av_ = 5.81) using a mobile phase with 80:20 (*v*/*v*) ACN:H_2_O at a flow rate of 0.600 nL/min. It was shown that %RSD values were less than 1%, which indicate that the developed column was suitable for nano-LC separations. Column-to-column reproducibility was investigated for ANM-8 column preparations on different days and from two different batches of ANM monomer, which were independently synthesized in our laboratory. These fabricated ANM-8 columns from two batches of the ANM monomer were prepared independently, and the *k* values of the analytes were estimated under the same mobile phase composition. The results are looking promising regarding the reproducibility. With a total of 30 different runs, %RSDs of 2.91, 2.12, and 1.66 were observed for uracil, toluene, and butyl benzene, respectively. %RSDs in the range of 1.5–3.0 indicate promising reproducible results for the monoliths, which were prepared at different times. The result shows that the developed monolith was capable for use in nano-LC. The separation efficiency was evaluated using a plot of plate height versus the linear flow velocity (van Deemter plot) whereby 80/20% (*v*/*v*) ACN:H_2_O was used as the mobile phase.

**Figure 3:**
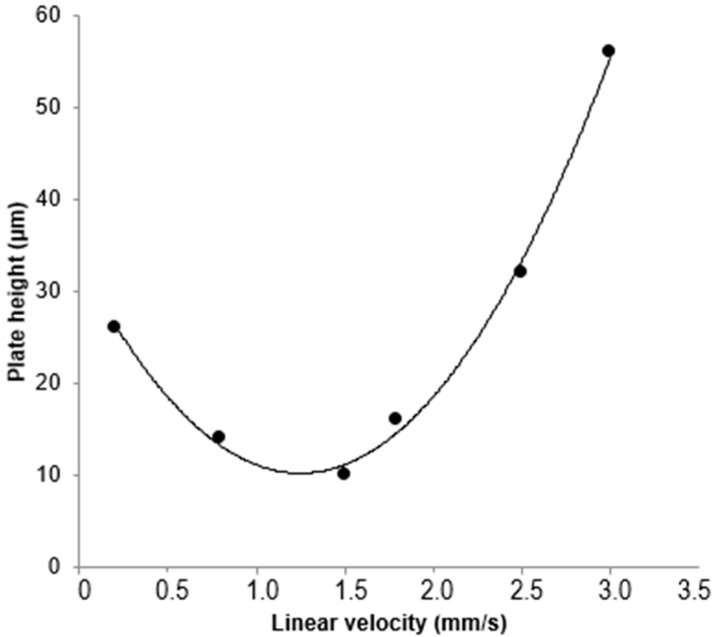
The effect of linear flow velocity on the plate height of ANM-8 monolithic column with butylbenzene.

As shown in Figure 3, the ANM-8 monolithic column yielded an average minimum plate height of 9 µm for uracil, which produced more than N ≈ 46.220 plates/m. The obtained results indicated that ANM-8 monolith could use a wide range of nano-liter flow rates.

### 2.2. Chromatographic Evaluation

Alkylbenzene homologous (ABs) as model nonpolar compounds are commonly used for reversed-phase (RP) monolithic column evaluation [15]. Five AB homologs were tested on the ANM-8 monolithic column in the range of 70–90% (*v*/*v*) ACN in the mobile phase at different nano-flow rates (e.g., 1000 nL/min, 800 nL/min, and 600 nL/min). The monolithic columns with different ANM contents (e.g., ANM-6,7,8 columns) were used for AB separation in the nano-LC system.

**Figure 4:**
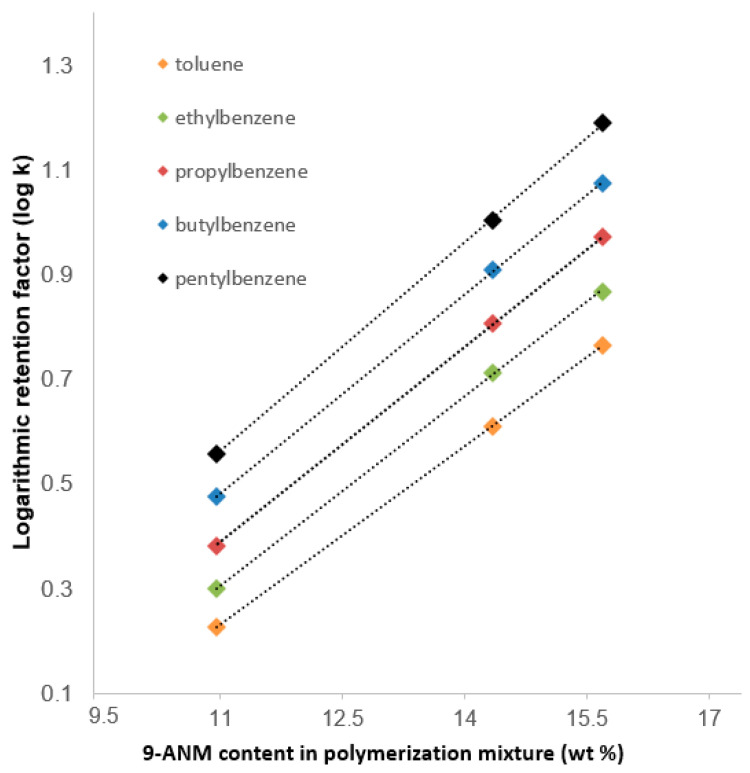
Logarithmic retention factor (log k) of alkylbenzenes versus ANM content in the polymerization mixture.

**Figure 5:**
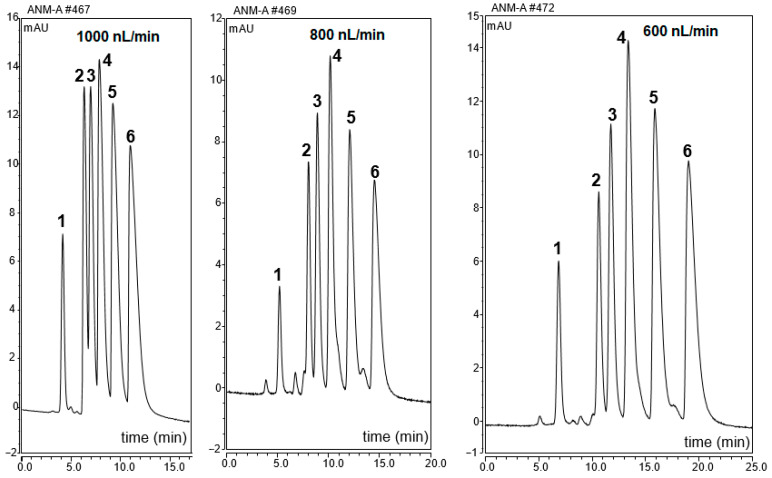
Chromatograms of alkylbenzene separation with the mobile phase (88/12% ACN/H_2_O *v*/*v*) using ANM-8 monolith at various flow rates. Order of peaks: (1) thiourea, (2) methylbenzene (MB), (3) ethylbenzene (EB), (4) propylbenzene (PB), (5) butylbenzene (BB), and (6) pentylbenzene PB.

Figure 4 shows the obtained results in terms of log *k* of AB versus ANM content. Increasing the ANM content in the polymerization mixture allowed more retention for ABs on the columns. The best result could be obtained using the ANM-8 column with the ANM content of 16.02%. Various ACN contents from 70 to 90% in the mobile phase were applied for AB separation using the ANM-8 column. The best separation was achieved using the mobile phase (ACN:H_2_O 88:12% *v*/*v*). The results are also typical of monolithic RP column, which was attributed to the logarithmic methylene group selectivity (log_αCH2_) [15]. Five ABs were well separated using the ANM-8 monolith with a higher content of ACN in the mobile phase (e.g., 80%, *v*/*v*) at different flow rates (see Figure 5). The obtained results showed that the ANM-8 monolithic column was a promising material for small solutes, indicating that the separation mechanism under RP chromatography conditions originated from ANM, which is consistent with the reported literature [16]. Considering both strong π-π stacking interactions originated from the ANM and electron-donating effect towards the aromatic solutes, we selected four polyaromatic hydrocarbons (PAHs) for further testing of the ANM-8 column, which may be promising behavior for peptide and protein separation.

**Figure 6:**
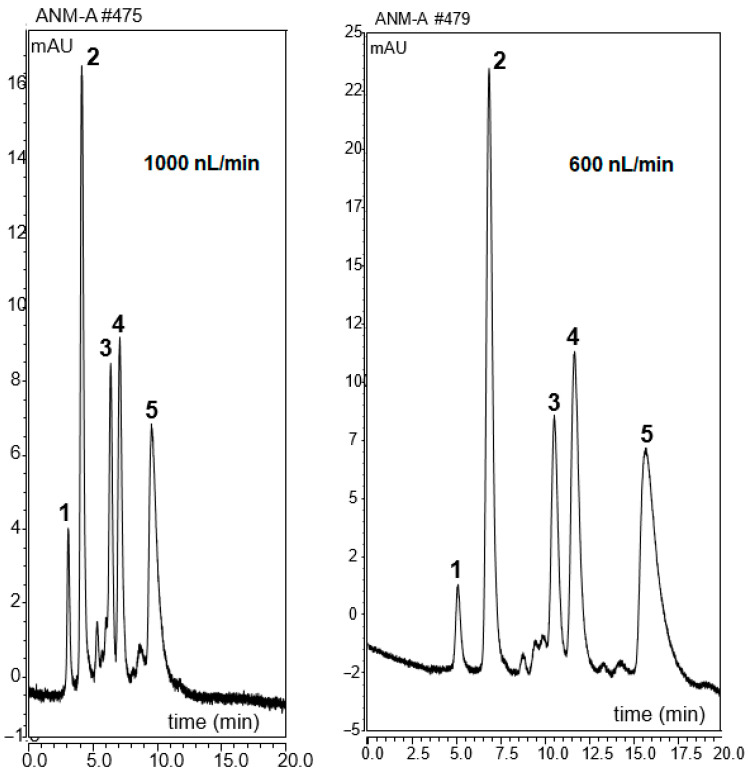
The chromatograms of PAH separation with the mobile phase (85/15% ACN/H_2_O *v*/*v*) using ANM-8 monolithic column at various flow rates. Detection wavelength, 220 nm; column length, 12 cm. Order of peaks: (1) thiourea, (2) naphthalene, (3) phenanthrene, (4) anthracene, and (5) pyrene.

ANM monomer is a very hydrophobic molecule, which may be promising for the preparation of hydrophobic monoliths. Hydrophobic interaction is key for protein separation, and protein hydrophobicity depends on both the 3D structure and amino acidic composition [17]. Therefore, the use of hydrophobic monolith is promising for both protein and peptide separation. In this respect, four PAHs, including naphthalene, anthracene, phenanthrene, and pyrene, were used for the evaluation of developed monolithic column hydrophobicity, which are more hydrophobic than ABs. PAHs were separated using the ANM-8 column with a high content of ACN in mobile phase with (ACN:H_2_O (85:15%, *v*/*v*)) at different nano-flow rates. The separation chromatogram of four PAHs was given in Figure 6. It shows that a good separation was obtained using the ANM-8 column, even if a high nano-flow rate was applied, which took less than 10 min. As shown here, promising hydrophobic interactions between column and PAHs take place under RP chromatography conditions, which indicate the developed monolith was suitable for the separation of compounds with different hydrophobicity, including peptides and proteins.

### 2.3. Biomolecule Separation

The separation of peptides was studied using an ANM monolith at different pHs in the mobile phase. Three different dipeptides, including Ala-Tyr, Gly-Phe, and L-carnosine, were separated on the ANM-8 column. It was shown that the baseline separation could not be obtained at a low pH (e.g., pH 3.5) since the retention of the peptides generally decreased with decreasing pHs of the mobile phase. This was due to the charged groups on the peptides showing no interaction with the monolith, and the separation was optimized at neutral pHs (e.g, pH 7.0). When the pH increased, a considerably peak broadening is observed for the tested peptides. Figure S1 shows the separation chromatogram of three dipeptides at different pH values. In this separation, the ANM-8 monolith displayed both strong hydrophobic retention and π-π interactions.

The peptides were well separated on the monolith using the mobile phase with 40/60 (*v*/*v* %) 50 mM phosphate buffer/ACN at pH 7.0, which indicated the suitability of the monolith for biomolecule separation.

**Figure 7:**
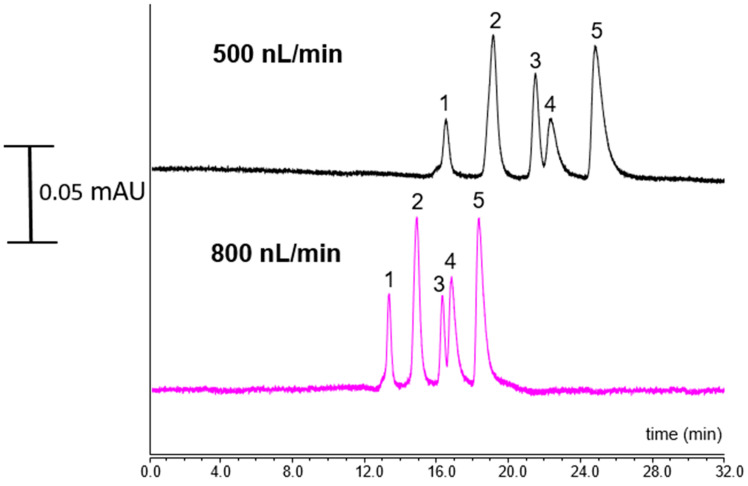
Chromatograms of five standard proteins using ANM-8 monolith. Mobile phases A: 5% ACN/95% H_2_O at 0.01% *v*/*v* TFA and mobile phase B: 95% ACN/5% H_2_O at 0.01% *v*/*v* TFA. Elution order of peaks: (1) RNase A, (2) Cyt C, (3) Lys, (4) Mb, and (5) α-Chym A.

The use of narrow columns provides several benefits in biomolecule separation, especially for a protein-centric (top-down) approach. The separation of proteins in very complex mixtures is a crucial issue in several disciplines such as in medical research. In our previous study, we applied a mobile phase containing 10% ACN/90% H_2_O at 0.1% *v*/*v* TFA as mobile phase A and 90% ACN/10% H_2_O at 0.1% *v*/*v* TFA as mobile phase B, linear gradient elution, 20–95% B in 3 min, retained 90% B for 5 min [18]. In the current study, the developed ANM-8 monolithic column was also applied for the separation of five standard proteins. The tested proteins were Mb (pI 6.88–7.33), Lys (pI 11.0), Cyt C (pI 10.0–10.5), α-Chym A (pI 8.75), and RNase A (pI 9.6). It was shown that a high resolving separation of the proteins with the ANM-8 monolith could be obtained at different flow rates (see Figure 7).

As shown here, the proteins were baseline separated at low flow rates using the ANM-8 monolith, indicating a good mass loadability. Protein hydrophobicity depends both on the dimensional structure with surface hydrophobicity and amino acid residues, which is an important issue in protein separation. These proteins include those that ranged from 104 to 241 amino acid residues [17], which affected the retention of the proteins. The elution order was shown as RNase A, Cyt C, Lys, My, and α-Chym. A polymer based monolith with 100 µm ID was prepared and applied for the protein separation [19]. A better separation performance could be achieved for the separation. The obtained results showed that the developed ANM monolith is capable for the separation of proteins, indicating a promising material for top-down proteome analysis.

### 2.4. Protein and Peptide Profiling

Innovations in miniaturized LC are continuing for achieving new levels of sensitivity and selectivity in biomolecule separation. In this sense, nano-LC is the most common tool for proteomics analysis [20]. The use of the reduced column inner diameter provides good sensitivity, offering several benefits in limited samples. In addition, when comparing the 4.6 mm ID column with the 100 µm ID column, the sensitivity up to 4000-fold may be obtained. The narrow columns also allow a high number of molecule identification in proteomics analysis, which is often challenging for small numbers of samples. Proteome samples are also extremely complex, which is a greatly challenging issue. In current study, the ANM-8 monolith with 25 cm length was applied for the protein and peptide profiling of MCF-7 cell lines using a gradient elution of % 2–35 B at a flow rate of 400 nL/min in nano-LC.

**Figure 8:**
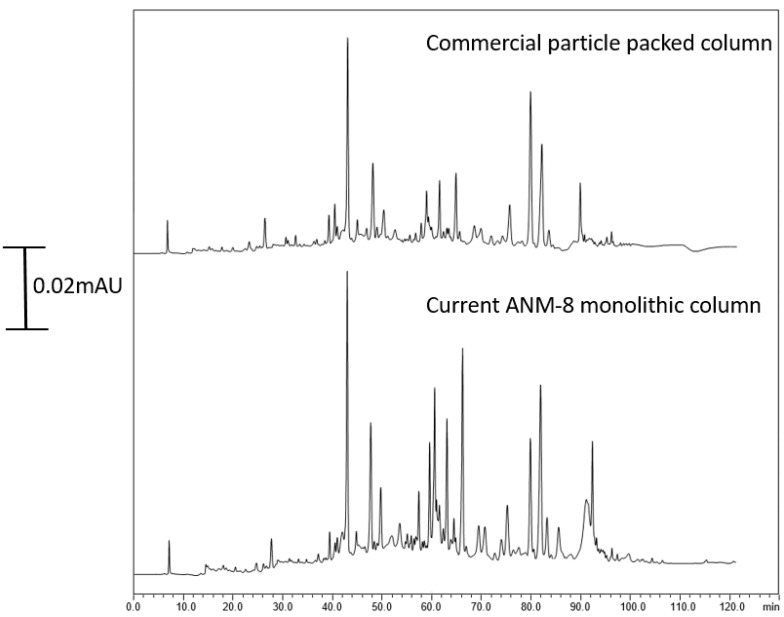
The protein and peptide profiling of MCF-7 cell line using both the ANM-8 monolith at 25 cm in length and commercial particle packed column. The chromatographic conditions are same.

The column length effected the peptide resolution while π-π interactions and strong hydrophobic retention played a crucial role in peptide retention. The developed column was also compared with a counterpart commercial nano-LC column using the same cell line. As shown in Figure 8, the better performance could be achieved using the ANM-8 monolithic column. These results showed that the developed ANM monolithic column could be a promising alternative for high resolving power for further protein and peptide profiling.

## 3. Materials and Methods

### 3.1. Chemicals and Reagents

The main monomer, 9-anthracenylmethyl methacrylate (ANM), was synthesized in our laboratory (see next section). Trimethylolpropane trimethacrylate (TRIM) was purchased from Sigma Aldrich (St. Louis, MO, USA) Homologous alky lbenzenes and polyaromatic hydrocarbons (PAHs) were purchased from Merck A.G (Darmstad, Germany). A trap column (PEPMAP 100 C18, 5UM, 0.3 × 5 mm Lot: 00850935) was used for pre-column extraction. A reversed-pahse column C18 (1.8 µm, 100 µm × 250 mm) was used for the evaluation of the developed column performance. Empty fused silica capillary column with 100 µm ID were purchased from BGB Analytik (Istanbul, Türkiye). Five intact proteins, including RNase A, Lys, Cyt C, Mb, and α-Chym A were purchased from Sigma Aldrich (St. Louis, MO, USA). Dipeptides, including Ala-Tyr, Gly-Phe, and L-carnosine were also purchased from Sigma Aldrich (St. Louis, MO, USA).

### 3.2. Instrumentation

NCS-3500RS Nano ProFlow (5041.0010A) nano-LC system Thermo Scientific Dionex was used for chromatographic separation and LC-UV experiments. The system included an autosampler with WPS-3000TPL RS and UV–Vis detector-3400RS with a 3 nL flow cell. Ultra-pure water was obtained using a Direct-Q^R^-3 from Millipore corporation (Billerica, MA, USA). Thin-layer chromatography (TLC) was used for the monitoring of the reaction process ANM monomer synthesis employing 0.25 mm thick precoated silica plates. A 400 (100) MHz Bruker spectrometer was used for obtaining ^1^H and ^13^C NMR spectra.

### 3.3. Cell Culture and Peptide Preparations

The preparation procedure of the proteome sample for MCF-7 cell lines was given in the Supplementary section (Figure S2).

### 3.4. ANM Monolithic Column Preparation

#### 3.4.1. Monomer Synthesis

**Figure 9:**
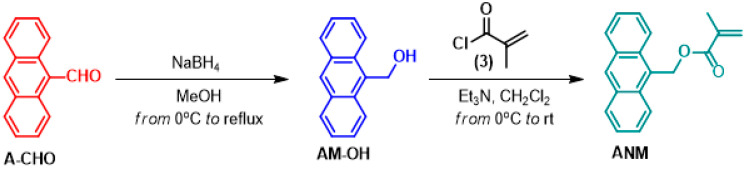
The reaction synthetic route 9-anthracenylmethyl methacrylate (ANM).

Figure 9 shows the reaction synthesization route for 9-anthracenylmethyl methacrylate (ANM). First, ANM was synthesized according to the published literature [21]. The related aryl-aldehyde (Ar-CHO, 1 equiv.) was dissolved in dry MeOH (50 mL) under nitrogen atmosphere. While the reaction is kept at 0 °C, NaBH_4_ (1.5 equiv. as mL) was added to the solution in portions for about 5 min.

The reaction mixture was stirred for 30 min, slowly warming to room temperature. Following this, the reaction mixture was heated to the reflux temperature of MeOH for 5–6 h. After checking with TLS whether the reaction was finished or not, the reaction mixture was cooled down to room temperature and diluted with HCl (5%, 100 mL). Following this, the white precipitate was collected by filter and dissolved into EtOAc; the organic solution was then washed with brine (3 × 20 mL) and after dried over sodium sulfate (Na_2_SO_4_), and filtered, and the solvent was evaporated in vacuo. The crude product was further purified via flash column chromatography with EtOAc/hexane (25%), and the target aryl-methyl alcohols (ArM-OHs) were obtained as follows. The 9-anthracenylmethanol (AM-OH, 87%) was obtained as a yellow solid. ^1^H-NMR (400 MHz, CDCl_3_): δ 8.41–8.49 (m, 3H, =CH), 8.02–8.06 (m, 3H, =CH), 7.49–7.59 (m, 4H, =CH), 5.69 (m, 2H, CH_2_). The synthesis of ANM was performed according to the published literature [22]. The 9-anthracenylmethanol (AM-OH, 1 equiv.) was dissolved in dry CH_2_Cl_2_ (50 mL); following this, triethylamine (Et_3_N) (1.5 equiv. as mL) was added to the solution. While the reaction is kept at 0 °C, the methacryloyl chloride (1.5 equiv. as mL) in CH_2_Cl_2_ (10 mL) was added dropwise for about 30 min via dropping funnel. The reaction mixture was stirred overnight, slowly warming to room temperature. After checking with TLC whether the reaction is finished or not, the reaction mixture was washed with 30 mL of aqueous sodium bicarbonate (NaHCO_3_), water, and finally with brine. Following this, the organic layer was dried over sodium sulfate (Na_2_SO_4_), and filtered, and the solvent was evaporated in vacuo. The crude product was further purified via flash column chromatography with CH_2_Cl_2_/hexane (25%), and the main monomer of ANM was obtained as a pale yellow solid (see Figure S3). ^1^H-NMR (400 MHz, CDCl_3_): δ 8.50 (s, =CH, 1H), 8.40 (d, *J* = 8.5 Hz, =CH, 2H), 8.03 (d, *J* = 8.5 Hz, =CH, 2H), 7.56–7.60 (m, 2H, =CH), 7.48–7.52 (m, 2H, =CH), 6.23 (s, 2H, O-CH_2_), 6.07 (s, 1H, C=CH), 5.52 (s, 1H, C=CH), 1.93 (m, 3H, CH_3_); APT (see Figure S4A). ^13^C-NMR (100 MHz, CDCl_3_): δ 18.61 (CH_3_), 59.38 (CH_2_), 124.27 (CH), 125.33 (=CH_2_), 126.29, 126.61, 126.83, 129.32, 129.37, 131.33, 131.60, 136.65, 167.85. (see Figure S4B). The data are compatible with the literature [23].

#### 3.4.2. In Situ Polymerization

The fused silica capillaries with 100 µm ID were prepared and silanized according to the published literature [12]. A polymeric solution, including ANM as the functional monomer 16.02% (*w*/*w*), TRIM as the crosslinker 25.0% (*w*/*w*), and AIBN (0.9 wt %) as initiator, was prepared for monolith preparation. Porogenic solvent was composed of THF (45.49%) and 1-dodecanol (15.49%). Table 1 shows the composition of the polymerization solutions used in the preparation of ANM monolithic columns. After stirring of the polymerization solution for 5 min, it was degassed in an ultrasonic bath for 5 min at room temperature. The polymer solution was injected into the silanized capillaries with 8 cm length. The polymerization was performed in a water bath for 18 h at 65 °C. The final monolith was washed with ACN:H_2_O (80:20 *v*/*v*) for 2 h at a flow rate of 250 nL/min.

### 3.5. Chromatographic Conditions

ProFlow nano-LC system was used for chromatographic experiments. Butylbenzene as a retained marker was used for the determination of plate numbers while thiourea was used as an avoid marker. All test compounds as the standard solutions were prepared in the range of 0.005–500 µg/kg. Standard intact proteins were in the range of 0.05–200 µg/kg, which were prepared and then stored in the refrigerator. Nano-LC protein separation with the ANM monolith was performed according to the published literature [18].

## 4. Conclusions

The nano-LC with new developed columns is a promising tool for both small molecule separation and the protein and peptide profiling of the MCF-7 cell line. In this study, ANM-based monolithic nano-columns were, for the first time, prepared and characterized. The polymerization solution was optimized in relation to both the contents of monomer and porogenic solvent. After chromatographic characterization, the prepared monolith was applied both to the peptide and five standard protein separations. The peptides were well separated at neutral pHs while proteins were baseline separated; both π-π interactions and hydrophobic retention took place in the retention mechanism. The developed monolith was further applied to the MCF-7 cell line to evaluate the protein and peptide profiling performance. The developed ANM monolithic column exhibited good results for the protein and peptide profiling of the MCF-7 cell line as it is a good complementary nano-LC separation media. The ANM monolith was also compared with a commercial particle-packed column using the MCF-7 cell line as a better separation performance could be obtained. It was shown that a new alternative nano-LC monolith could be developed, which was also suitable for the protein and peptide profiling of the biological samples.

## Figures and Tables

**Table 1:** Composition of the polymerization solutions used for ANM monolith preparation.

Column	Monomers (Molar Ratio)	Porogen (µL)	R^2 a^	Specific Surface Area (m^2^/g)	Permeability (×10^−14^)
	ANM: TRIM	THF:Dodecanol			
ANM-1	1:2	100:300	No back pressure	-	-
ANM-2	1:2	200:300	No back pressure	-	-
ANM-3	1:2	200:200	Low back pressure	-	-
ANM-4	1:2	300:100	0.9964	-	-
ANM-5	1:2	400:100	0.9987	102.7	8.72
ANM-6	1:3	400:100	0.9991	-	-
ANM-7	1:3.2	400:100	0.9995	-	-
ANM-8	1:3.6	400:100	0.9998	288.4	2.22
ANM-9	1:3.8	400:100	Low flow	-	-
ANM-10	1:4	400:100	White jel	-	-

- No calculation; ^a^ linear relationship between flow rate and the resulting backpressure of relevant monolith.

## Data Availability

All figures and data used to support this study are included within this article.

## Supplementary Materials

The following supporting information can be downloaded at https://www.mdpi.com/article/10.3390/ijms252413646/s1.
