# Supplementary material for: Nano-LC with New Hydrophobic Monolith Based on 9-Antracenylmethyl Methacrylate for Biomolecule Separation
Source: Int J Mol Sci. 2024 Dec 20;25(24):13646. doi: 10.3390/ijms252413646 (PMC11728373; doi:10.3390/ijms252413646)
Supplement: Supplementary file 1 [file ijms-25-13646-s001.zip › ijms-3318310-supplementary.pdf]

Supporting Information for

Nano-LC with new hydrophobic monolith based on 9-antracenylmethyl methacrylate for biomolecule separation

Cemil Aydoğan* [1,2,3], Sarah Alharthi[4]

[1]Food Analysis and Research Laboratory, Bingöl University, 12000 Bingöl, Türkiye
[2]Department of Food Engineering, Bingöl University, 12000 Bingöl, Türkiye
[3]Department of Chemistry, Bingöl University, 12000 Bingöl, Türkiye
[4]Department of Chemistry, College of Science, Taif University, P.O. Box 11099 Taif, Saudi Arabia

*Corresponding author

caydogan@bingol.edu.tr

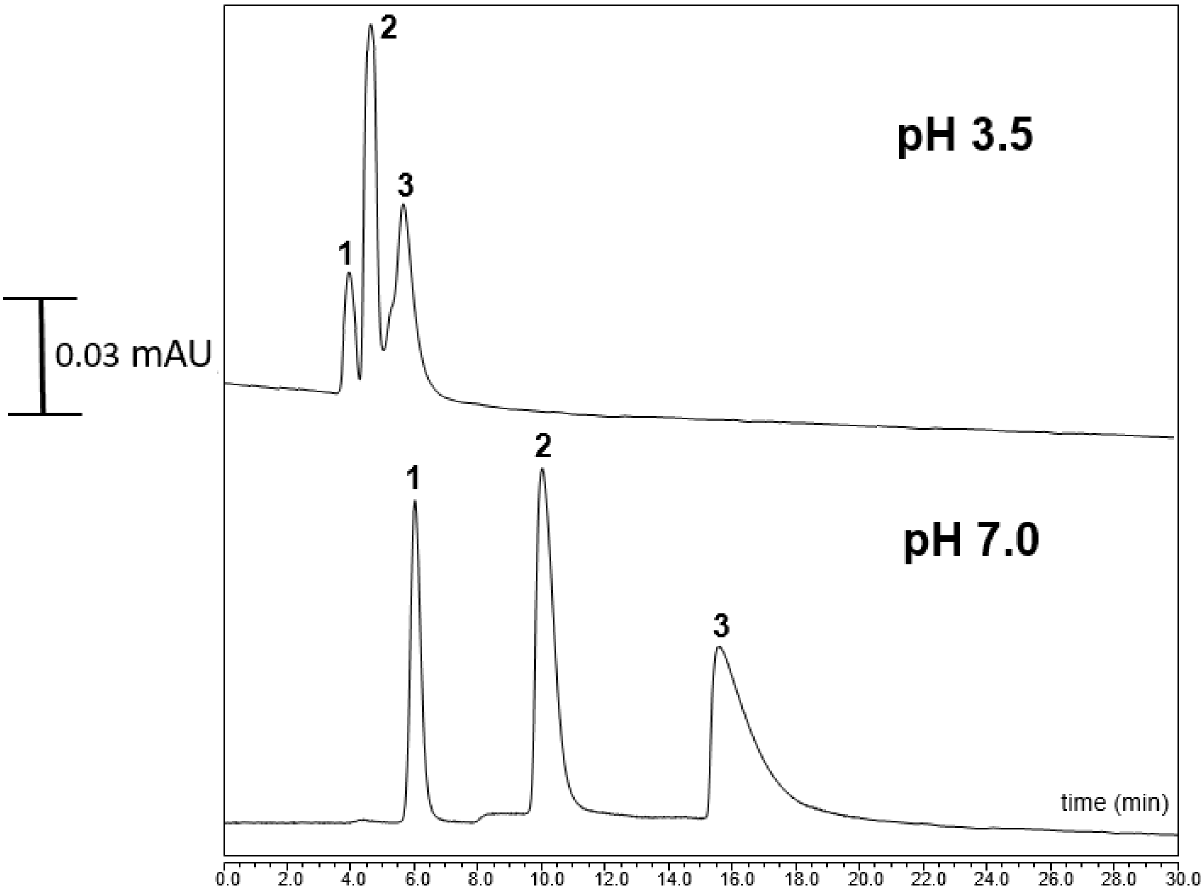

Figure S1 The chromatograms of peptide separation with the mobile phase (40/60 (v/v %) 50 mM phospahte buffer/ACN at pH 7.0) using ANM-8 monolithic column. at different pH values. detection wavelegth; 214 nm, order of peaks; (1) L-carnosine (2) Ala-Tyr (3) Gly-Phe.

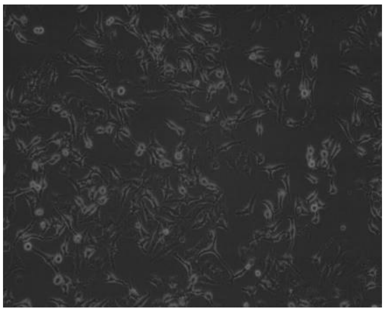

Figure S2: *MCF-7 Cell line*

MCF-7 cells were grown according to ATCC protocols to 80-90% confluence in order to obtain peptides for chromatograpghic analyses. Cultured cells were washed twice with ice-cold phosphate-buffered saline (PBS) and lysed by 8 M urea, 50 mM Tris-HCl, pH 8.0 and 1% by volume Protease Inhibitor Mix (no EDTA is used) (Sigma-Aldrich) on ice. The cell lysate were collected and centrifuged at 20,000 g for 20 min at 4°C. Protein concentrations were measured using Bradford assay (Bio-Rad, Hercules, CA). The protein samples were reduced by 10 mM dithiothreitol (DTT) and incubated at 37 ◦C for 1h. After alkylated by Chloroacetamide, the protein sample was diluted with five volume 50 mM NH_4HCO_3. Protein sample were digested by Trypsin (0.2mg/mL) 1:100 (w/w) and the digest mixture were incubated overnight at 37°C. Peptide samples were centrifuged at 5000 g for 15 min and then, desalted with Sep-Pak C18 column. Each sample was loaded onto a Sep-Pak C18 column, and eluted by 1 mL 50% ACN/0.1% FA in water and stored at −80 °C until further use.

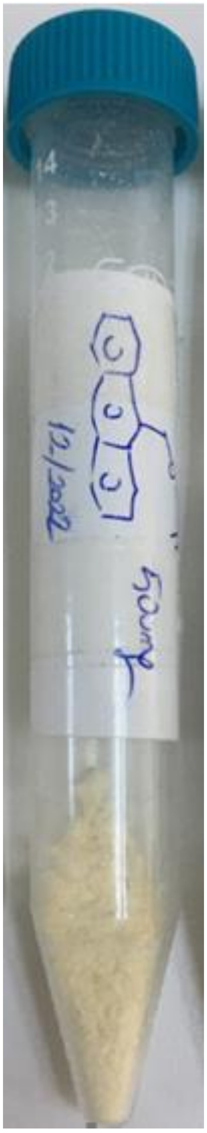

Figure S3: The obtained 9-ANM monomer as pale yellow solid

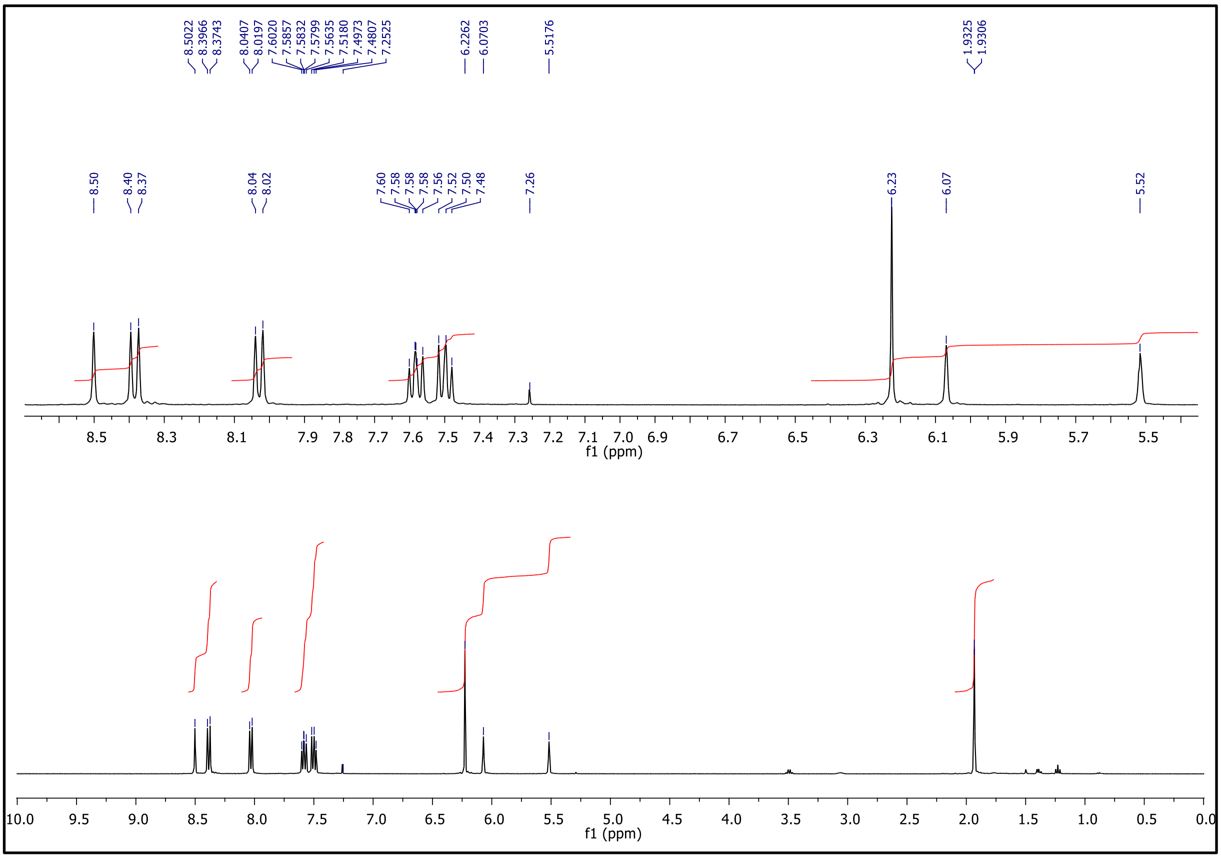

Figure S4-A ^{1}H-NMR (400 MHz) for 9-ANM monomer

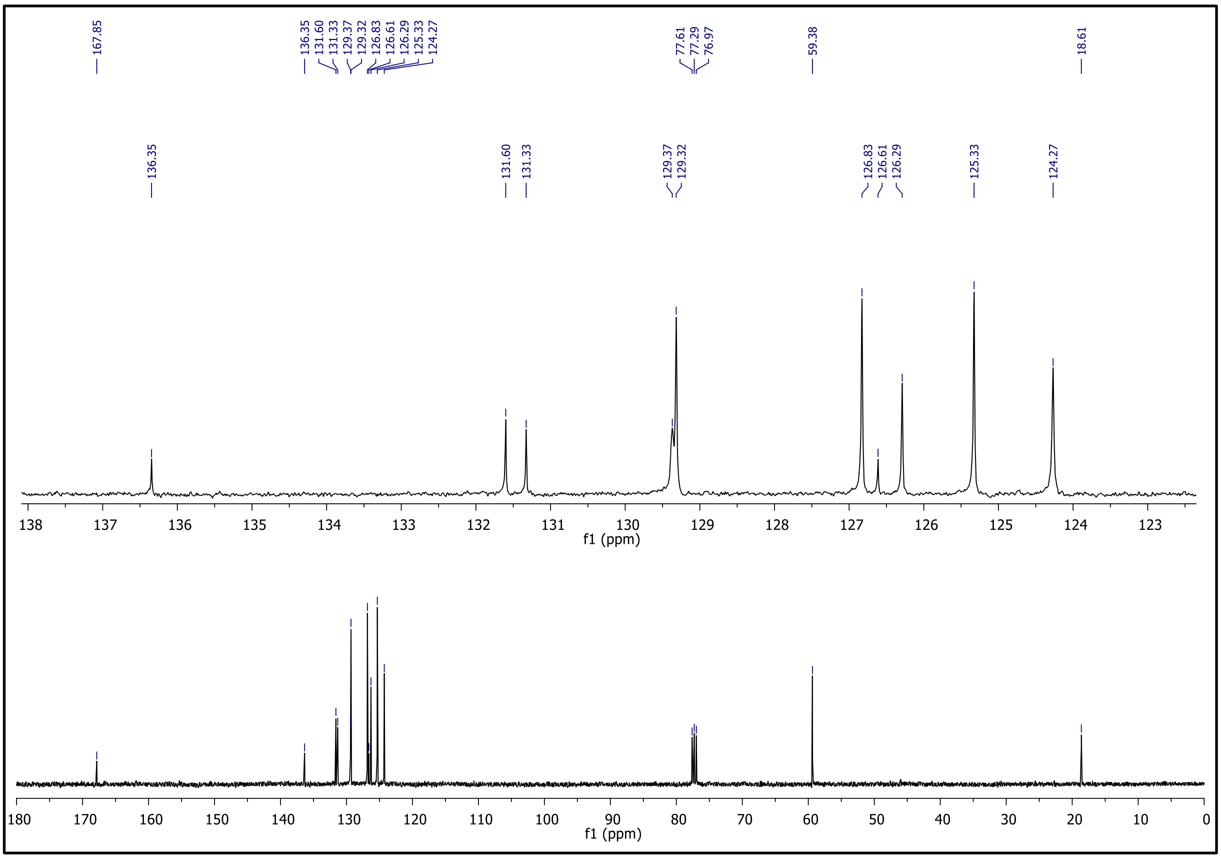

Figure S4-B ^{13}C-NMR (100 MHz) ($CDCl_3$) for 9-ANM monomer.
